# Diffusion of Ofloxacin in the Endocarditis Vegetation Assessed with Synchrotron Radiation UV Fluorescence Microspectrocopy

**DOI:** 10.1371/journal.pone.0019440

**Published:** 2011-04-29

**Authors:** Eric Batard, Fréderic Jamme, Sandrine Villette, Cédric Jacqueline, Marie-France de la Cochetière, Jocelyne Caillon, Matthieu Réfrégiers

**Affiliations:** 1 Université de Nantes, EA3826 Thérapeutiques cliniques et expérimentales des infections, Nantes, France; 2 Disco beamline, Synchrotron Soleil, Saint-Aubin, France; 3 CEPIA, Institut National de la Recherche Agronomique, Nantes, France; 4 Centre de Biophysique Moléculaire UPR 4301, Centre National de la Recherche Scientifique, Orléans, France; Aga Khan University, Pakistan

## Abstract

The diffusion of antibiotics in endocarditis vegetation bacterial masses has not been described, although it may influence the efficacy of antibiotic therapy in endocarditis. The objective of this work was to assess the diffusion of ofloxacin in experimental endocarditis vegetation bacterial masses using synchrotron-radiation UV fluorescence microspectroscopy. Streptococcal endocarditis was induced in 5 rabbits. Three animals received an unique IV injection of 150 mg/kg ofloxacin, and 2 control rabbits were left untreated. Two fluorescence microscopes were coupled to a synchrotron beam for excitation at 275 nm. A spectral microscope collected fluorescence spectra between 285 and 550 nm. A second, full field microscope was used with bandpass filters at 510–560 nm. Spectra of ofloxacin-treated vegetations presented higher fluorescence between 390 and 540 nm than control. Full field imaging showed that ofloxacin increased fluorescence between 510 and 560 nm. Ofloxacin diffused into vegetation bacterial masses, although it accumulated in their immediate neighborhood. Fluorescence images additionally suggested an ofloxacin concentration gradient between the vegetation peripheral and central areas. In conclusion, ofloxacin diffuses into vegetation bacterial masses, but it accumulates in their immediate neighborhood. Synchrotron radiation UV fluorescence microscopy is a new tool for assessment of antibiotic diffusion in the endocarditis vegetation bacterial masses.

## Introduction

Bacterial endocarditis is a severe infection developing at the surface of the cardiac valvular apparatus. The endocarditis lesion, the so-ca lled vegetation, is mainly composed of bacterial masses embedded in a platelet and plasma proteins matrix [1]. The diffusion of antibiotics in the endocarditis vegetation is a key determinant for their activity in this difficult-to-treat infection [2]. As the endocarditis vegetation is an avascular lesion, antibiotics diffuse into it from its periphery to its centre [3]. Similarly, antibiotics diffuse from the periphery of bacterial masses to their centre. The diffusion of antibiotics in the experimental endocarditis vegetation has been assessed by autoradiography for various antibiotics [3], [4], [5], [6]. However, due to autoradiography poor spatial resolution, the diffusion of antibiotics within the vegetation bacterial masses has not been studied. This is a matter of concern, because poor antibiotic diffusion in vegetation bacterial masses may be a risk factor for treatment failure.

Fluorescence microscopy has previously been used to study the diffusion of tetracyclins in bacterial biofilm and uninfected bone thanks to their particular fluorescence properties compatible with epifluorescence microscopy and confocal scanning laser microscopy [7], [8]. Similar experiments have been reported with tagged vancomycin and daptomycin [9], [10]. Synchrotron-radiation UV fluorescence microspectroscopy increases the field of application of fluorescence microscopy to probes that can be excited down to 200 nm. Many aromatic and phenolic compounds can therefore be mapped without any external probes [11], [12]. The ultraviolet radiation emitted from a bending magnet of the synchrotron is monochromatized and collected toward a full UV microscope, allowing microscopic acquisition at high spatial resolution - typically at the µm scale or lower.

The diffusion of ofloxacin in the endocarditis vegetation has not been reported. Ofloxacin has interesting autofluorescence properties, with emission wavelengths ranging from 400 to 600 nm after excitation at 275 nm [13]. The objective of this work is to assess the diffusion of ofloxacin in endocarditis vegetation bacterial masses using synchrotron radiation UV fluorescence microspectroscopy.

## Results

### Fluorescence of ofloxacin in PBS and serum

**Figure 1:**
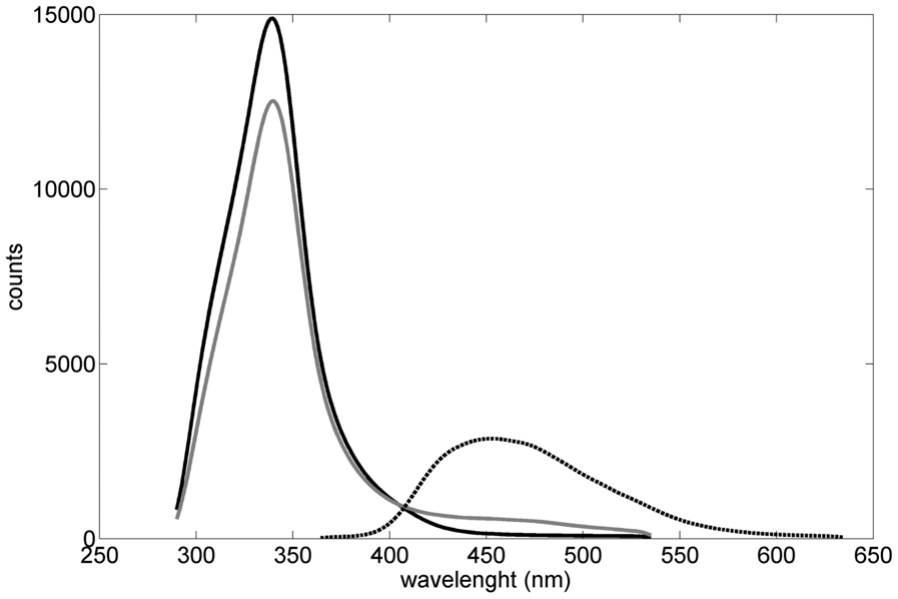
Fluorescence of ofloxacin in PBS (dot), ofloxacin in rabbit serum (black solid), and rabbit serum without ofloxacin (gray solid). Ofloxacin concentrations in PBS and serum were respectively 140 and 212 µmom/L (51 and 76 mg/L). Serum had a high intrinsic autofluorescence at 340 nm.

Ofloxacin in PBS (pH 7.4) showed a maximal fluorescence peak at 460 nm (range 390–600 nm) as shown in Figure 1. Ofloxacin-treated rabbit sera showed higher fluorescence intensities between 390 and 540 nm than control sera, with a maximum at 460 nm (Figure 1). The relationship between the 390–540 nm integrated peak area and ofloxacin serum concentration was assessed in 6 rabbit sera (ofloxacin serum concentrations ranging from 0 to 212 µmol/L, i.e. from 0 to 76 mg/L). The 390–540 nm peak area increased linearly with the ofloxacin serum concentration (Pearson r = 0.95, p = 0.004), thus showing a good correlation between serum fluorescence and ofloxacin concentration.

### Serum and vegetation ofloxacin concentrations

For rabbits treated by 150 mg/kg ofloxacin, ofloxacin mean ± SD concentrations were 170±16 µmol/L (61±6 mg/L) in serum and 241±27 nmol/g (87±10 µg/g) in vegetation.

### Comparison of control and ofloxacin vegetation spectra

Spectra extracted from vegetation maps of 3 ofloxacin-treated animals and 2 control rabbits were stratified on the 339 nm peak intensity by treatment status (control or ofloxacin), and by tissue class (bacterial mass or surrounding vegetation tissue). Then, selected spectra (control, n = 962; ofloxacin-treated, n = 962) were analyzed by Principal Component Analysis (PCA).

**Figure 2:**
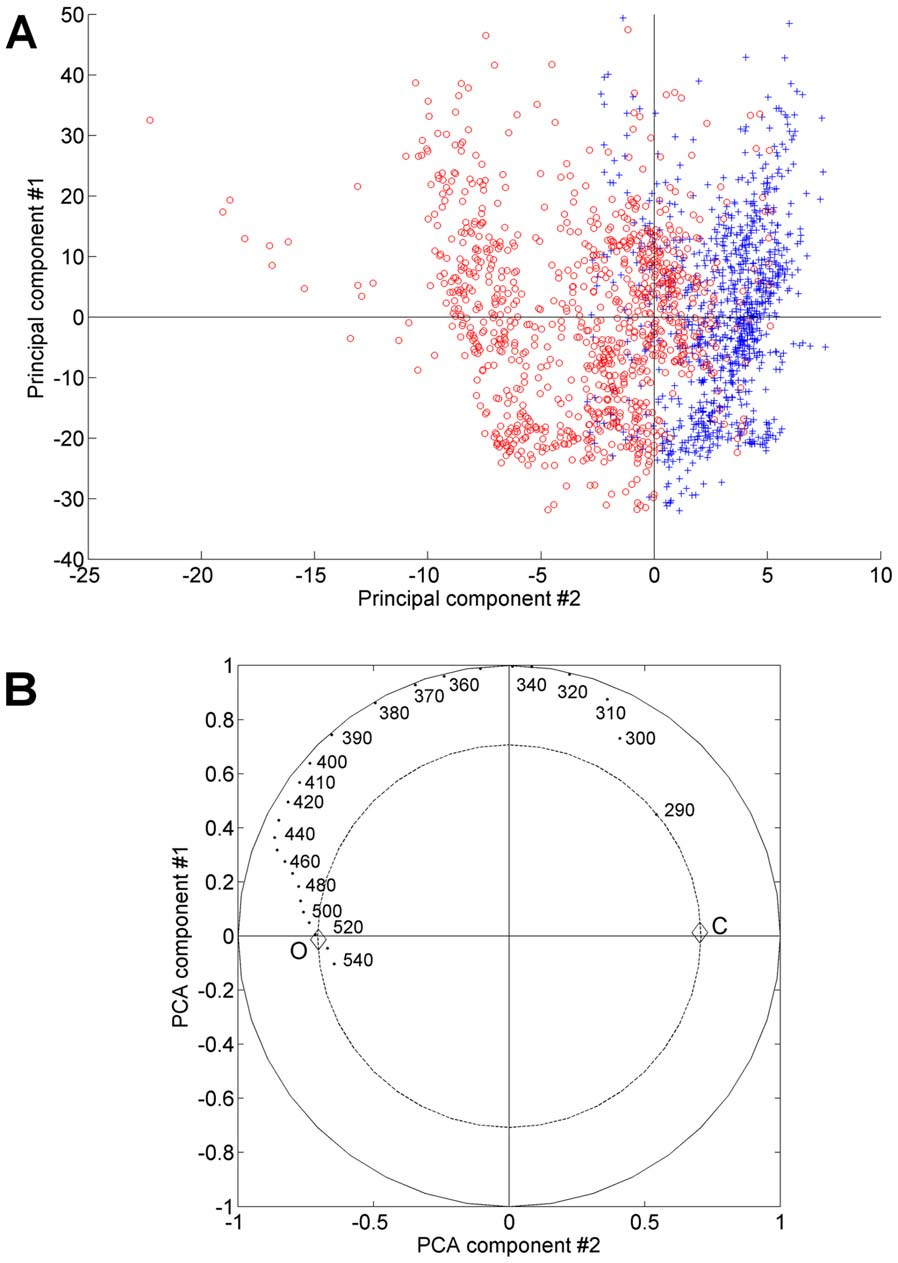
Principal component analysis of control and ofloxacin-treated vegetation spectra. Score plot of first (PC1) and second (PC2) principal components (A). Control (blue +) and ofloxacin-treated (red o) spectra were respectively associated with positive and negative PC2 scores. Correlation loading plot of PC1 and PC2 (B). Correlation of wavelengths with PC1 and PC2 scores are shown for wavelengths ranging from 290 to 540 nm. The outer ellipse and inner ellipse indicate 100% and 50% explained variance respectively. C = control; O = ofloxacin.

The first and second principal components (PC1 and PC2) accounted respectively for 89.5% and 7.8% of the total spectral variance. The score plot of PC1 and PC2 showed that the PC2 discriminated control and ofloxacin spectra (Figure 2A). The correlation loading plot showed that spectra with lower PC2 scores (i.e. ofloxacin spectra) were associated with higher values between 390 and 540 nm, and lower values at 290 nm (Figure 2B).

**Figure 3:**
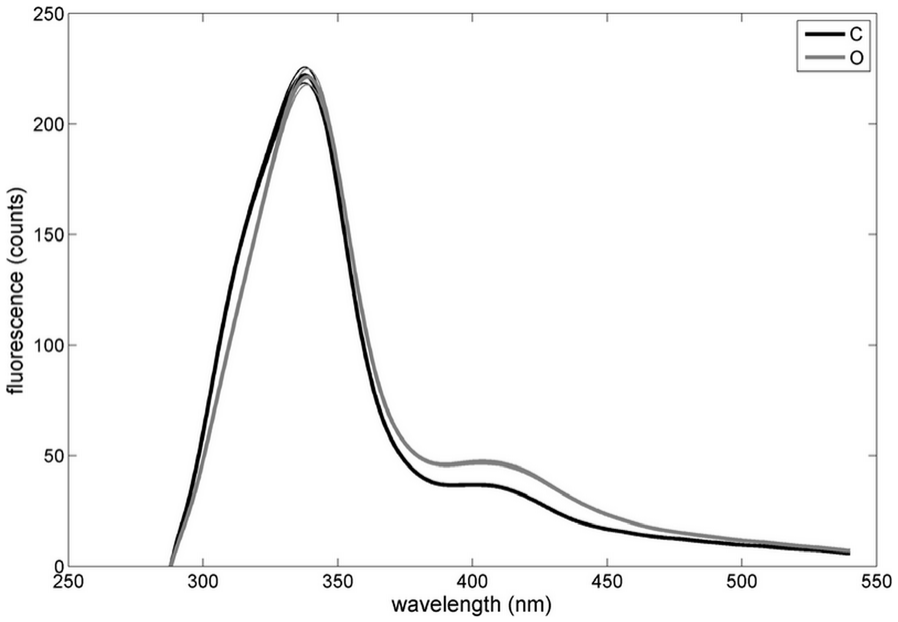
Mean spectra of untreated (gray) and ofloxacin (black) treated vegetations. Bold and thin lines represent respectively mean and borders of the 95% confidence interval. Confidence interval border lines may superimpose on mean lines.

After examination of mean control and ofloxacin spectra (Figure 3), we integrated separetely spectral areas between 390 and 440 nm, and between 440 and 540 nm (Table 1). Control and ofloxacin spectra were then compared for each tissue class. Ofloxacin increased significantly fluorescence between 390 and 440 nm, and between 440 and 540 nm, both in bacterial masses and in surrounding vegetation (Table 1).

**Table 1:** Fluorescence of control and ofloxacin vegetations.

Spectral range (nm)		390–440 ^a^	440–540 ^a^	510–560 ^b^
**Bacterial masses**	**Control**	774 (743–804)^c^	2879 (2810–2946)^c^	3824 (3745–3904)^c^
	**Ofloxacin**	855 (809–902)^c^	3694 (3588–3799)^c^	9828 (9782–9873)^c^
**Surrounding tissue**	**Control**	452 (431–473)^c^	2151 (2114–2189)^c^	5061 (5013–5109)^c^
	**Ofloxacin**	878 (819–939)^c^	2955 (2869–3940)^c^	9824 (9790–9859)^c^

Mean (95% confidence interval) peak areas (arbitrary units) were measured using spectral (a) and full-field (b) fluorescence microscopes. Differences between control and ofloxacin (c) were all significant (Mann-Whitney test, *P*<0.0001 for all comparisons).

### Spatial distribution of the 390–440 nm peak area

**Figure 4:**
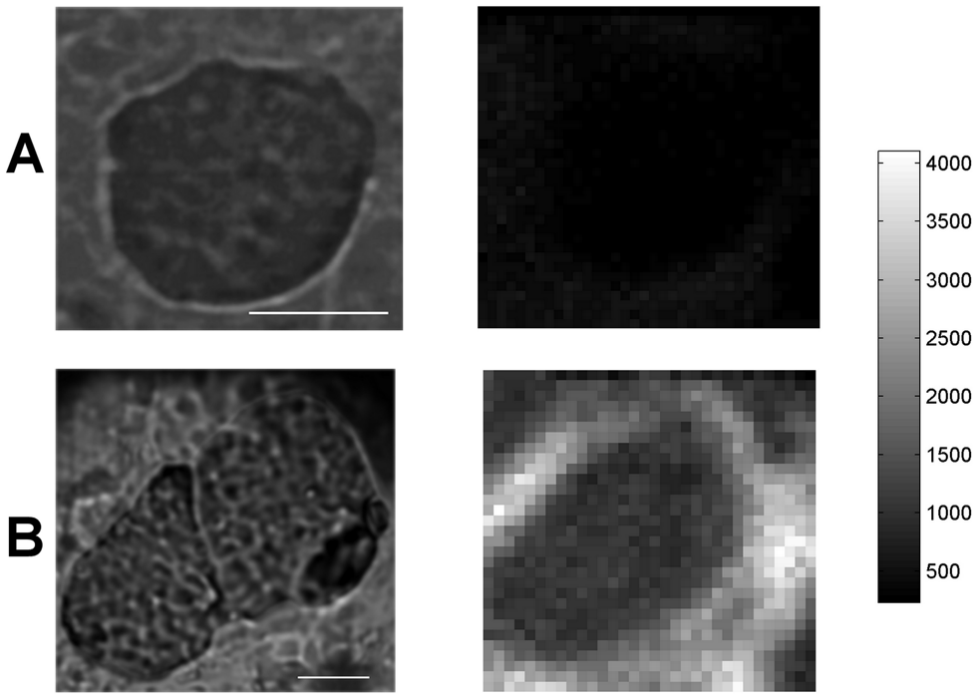
Transmission image (left) and maps of the 390–440 nm peak area (right) of control (A) and ofloxacin treated (B) vegetation maps. The grayscale was the same for both fluorescence maps. White bar = 10 µm.

In order to assess the diffusion of ofloxacin from surrounding vegetation into bacterial masses, maps of the 390–440 nm peak area were obtained from preprocessed spectra. Of note, spectra were not selected on the basis of their 339 nm band value as we did for PCA and previous statistics. Therefore, fluorescence intensities of these maps were different from values reported in Table 1. Two typical examples of control and ofloxacin-treated rabbits are shown in Figure 4. Maps of ofloxacin-treated vegetations showed that higher fluorescence values located in the vegetation tissue immediately next to the bacterial mass, suggesting that ofloxacin accumulated in the neighborhood of bacterial masses. Furthermore, fluorescence was uniformly distributed in the ofloxacin-treated bacterial masses.

### Spatial distribution of fluorescence between 510 and 560 nm

**Figure 5:**
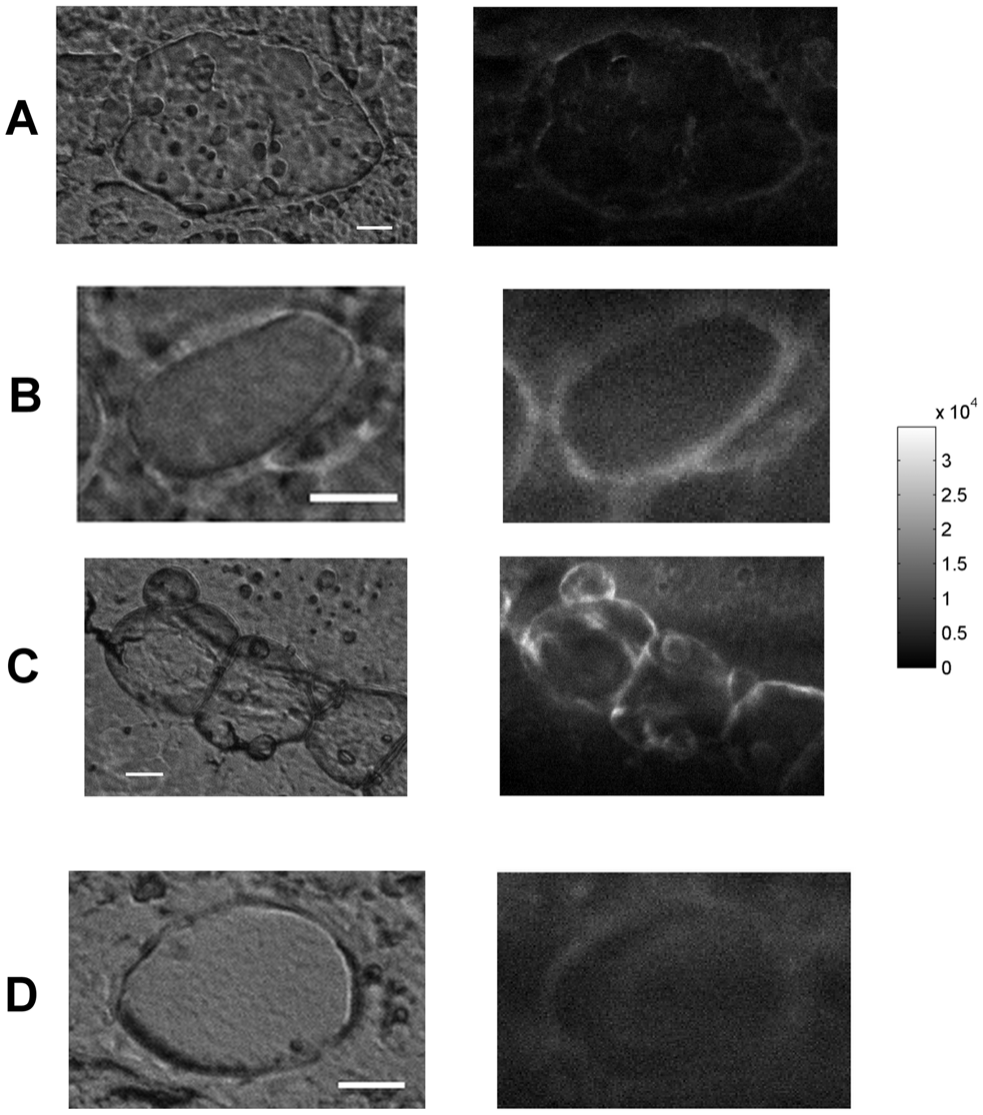
Fluorescence of control (A) and ofloxacin-treated (B,C,D) vegetation in the 510–560 nm range. Transmission (left) and fluorescence (right) images. The bacterial masses imaged on maps B, C and D located respectively in the intermediate, peripheral and central areas of the tissue specimen. The grayscale was the same for all maps. Fluorescence intensity values (median [range]) for A, B, C and D maps were respectively 2624 [0–14560], 8160 [1168–23376], 6736 [0–34928], and 6128 [432, 14640]. Each ofloxacin map intensity was significantly different from the control map (Mann-Whitney test, p<0.0001 for all). White bar = 10 µm.

The full field microscope collected fluorescence in the 510–560 nm spectral range (Figure 5). Fluorescence intensity was consistently lower for control tissue than for ofloxacin-treated animals. In a sample of 12 maps including more than 250,000 pixels, the mean (95% CI) fluorescence of control and ofloxacin maps were respectively 4953 (4936–4971) and 10306 (10293–10319), the difference being significant (*P*<0.0001). A subsample of >35000 of these pixels was classified as either bacterial mass or surrounding vegetation tissue (Table 1). The fluorescence of bacterial masses was significantly higher for ofloxacin than for control rabbits (*P*<0.0001). Similarly, the fluorescence of surrounding vegetation was significantly higher for ofloxacin than for control rabbits (*P*<0.0001). Altogether, this data showed that ofloxacin increased fluorescence between 510 and 560 nm, both in bacterial masses and in surrounding vegetation.

Fluorescence of ofloxacin bacterial masses and surrounding vegetation were very similar, indicating that ofloxacin diffused from the surrounding tissue into bacterial masses (Table 1). This can also be seen on typical images shown in Figure 5, which additionnally demonstrate that fluorescence inside bacterial masses was homogeneous.

Furthermore, the highest fluorescence intensities were observed for bacterial masses that located close to the border of the vegetation (Figure 5C). In contrast, bacterial masses in the central area of the vegetation showed lower intensity values (Figure 5D). This fluorescence gradient between peripheral and central areas of the tissue specimen suggests an ofloxacin concentration gradient in the endocarditis vegetation.

**Figure 6:**
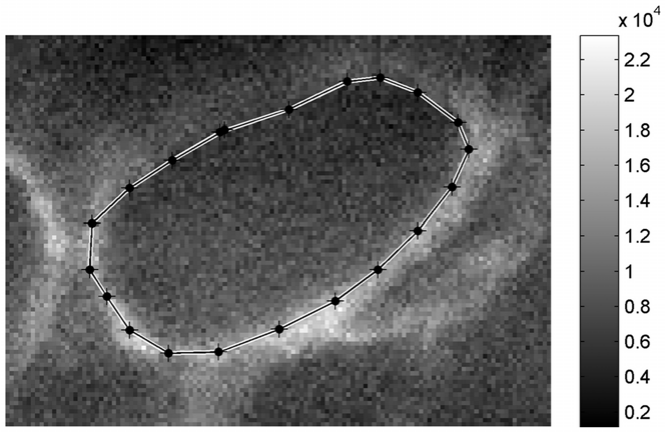
Fluorescence inside and next to an ofloxacin-treated bacterial mass. The position of the bacterial mass border was superimposed on the fluorescence map of the 510–560 nm range. For transmission image, see figure 5B.

The Figure 6 details the fluorescence of the same ofloxacin-treated bacterial mass as shown in Figure 5B. The maximal fluorescence intensities concentrated in the immediate neighborhood of the bacterial mass, showing that ofloxacin accumulates around it.

## Discussion

This study demonstrates that synchrotron-radiation UV fluorescence microspectroscopy detects ofloxacin in the endocarditis vegetation despite the autofluorescence of vegetation itself. The vegetation autofluorescence showed emission peaks at 339 and 410 nm, respectively due to tryptophan and NADH [11], [12]. The tryptophan band was lower in bacterial masses than in surrounding vegetation tissue, suggesting a lower protein content in bacterial masses, as we previously observed using infrared microspectroscopy [14].

Ofloxacin increased fluorescence intensities between 390 and 540 nm both in serum and in vegetation, and between 510 and 560 nm in vegetation. We do not claim that fluorescence allows to assess exactly the tissue or serum concentration of ofloxacin, but experiments on serum suggest that tissue fluorescence between 390 and 540 nm was roughly proportional to the ofloxacin concentration.

Two deep UV microscopes have been used in this study. The first microscope collected fluorescence spectra of vegetations, and allowed us to show that control and ofloxacin treated vegetations had different fluorescence patterns between 390 and 540 nm. Map acquisition with this microscope typically spends 1 h for a 80×80 µm^2^ map, and optical design yields a maximal sensitivity for wavelengths ranging from 300 and 450 nm. The second, full-field microscope gives no spectral information, but global fluorescence intensities for a given spectral range (510–560 nm in this study). Advantages of this second microscope are (i) high sensitivity (about 1000-fold the sensitivity of the spectral microscope), especially in the >500 nm range, and (ii) very short acquisition time (about 1 s for a 200×200 µm^2^ area). Hence the two fluorescence microscopes used in this study were complementary. In contrast with autoradiographic studies, synchrotron-radiation UV fluorescence microscopy allows the use of non-tagged drugs and assessments at multiple treatment time points. To be eligible to synchrotron-radiation UV fluorescence microspectroscopic studies, antibiotics should emit fluorescence light after excitation between 200 and 400 nm, with minimal interference with vegetation autofluorence emission spectra.

Our experiments were designed to demonstrate that synchrotron radiation UV fluorescence microspectrocopy detects ofloxacin in vegetation bacterial masses after a single injection. To maximize fluorescence detection, we administered a 150 mg/kg dose of ofloxacin, higher than doses previously used for experimental study of ofloxacin activity [15], [16]. As our model did not intend to reproduce the diffusion of ofloxacin in bacterial masses after a multiple human-like doses regimen, our results should not directly be extrapolated to what happens in patients.

Ofloxacin accumulates in the immediate neighborhood of vegetation bacterial masses, although this gradient may equilibrate in longer experiments. Furthermore, our work shows that ofloxacin diffuses into vegetation bacterial masses as soon as 30 minutes after the end of an unique injection. The diffusion of antibiotics in the endocarditis vegetation bacterial masses has not been previously reported, although poor diffusion is a potential risk factor for antibiotic therapy failure. The diffusion in vegetation bacterial masses may vary between antibacterial agents, thus influencing their antibacterial activity in endocarditis. Furthermore, diffusion of antibiotics in vegetation bacterial masses may be influenced by bacterial factors (e.g. biofilm or capsule production), and by antibacterial therapy modalities (e.g. injection number and duration). Using the example of ofloxacin, we showed here that synchrotron-radiation UV fluorescence microspectroscopy is a new tool to study the diffusion of antibacterial agents in infectious tissues and opens a new field of research in antibacterial chemotherapy.

## Methods

### Ethical statement

Animal experiments were carried out in accordance with European Commission Directive 86/609/EEC, and were approved by the committee of animal ethics of the University of Nantes (approval C44015). All surgery was performed under ketamine and diazepam anesthesia, and all efforts were made to minimize suffering.

### Experimental streptococcal rabbit aortic endocarditis

New Zealand white rabbits (weight: 2.0 to 2.7 kg) were kept in cages with free access to food and water. A polyethylene catheter was inserted into the left ventricle via the carotid artery and left in place throughout the experiment. *Streptococcus sanguis* was cultured overnight in Mueller-Hinton broth. After dilution in NaCl 9 g/l to obtain 5.10^7^ CFU/ml, a 1- ml suspension was inoculated into the ear vein 48 h after catheterization. Five days after inoculation, 3 rabbits received a 150 mg/kg dose of ofloxacin during a 60-minute intravenous injection and 2 animals received 0.9% saline. Rabbits were euthanized 30 minutes after the end of perfusion by IV injection of thiopental. Blood was taken by cardiac puncture when rabbits were euthanized. After centrifugation (5000 xg for 10 min at 4°C), sera were stocked at −80°C. Vegetations were excised and cryo-sectionned in 8-µm-thick slices that were deposited on UV transparent slides (CeNing Optics, Fujian, China). Contiguous slices were stained with Hematoxylin and Eosin to localize bacterial masses within the vegetation.

Three additional rabbits were treated with 150 mg/kg ofloxacin to assess ofloxacin concentration in homogeneized vegetations. To assess the relationship between serum fluorescence and ofloxacin concentration, 4 rabbits were treated with lower doses of ofloxacin (20 and 120 mg/kg). Ofloxacin concentrations in serum and in homogeneized vegetations were assessed by bioassay. Standard curves for homogeneized vegetation and in sera were constructed respectively in PBS and in serum, as previously described [17].

### UV Fluorescence Microspectroscopy

Bacterial masses were localized under visible light in confrontation with the HE stained contiguous slices. Hence, each pixel, and consequently each fluorescence spectrum, could be classified in either bacterial mass or surrounding tissue. Two UV microscopes directly coupled to the synchrotron beam at DISCO Beamline, Synchrotron SOLEIL, were used. The first, spectral microscope was constructed around an Olympus IX71 inverted microscope as previously described and used with a Zeiss Ultrafluar 40x objective [11]. It collected consecutively fluorescence spectra every 2 µm after excitation at 275 nm. Collection time was 2 s for each spectrum, and detection range was 285–550 nm. Typical maps ranged 80×80 µm^2^.

The second, full field imaging system was a Zeiss Axio observer microscope with a Zeiss Ultrafluar 40x objective. Dichroic mirrors with 50% transmission/reflexion at 300 nm (Omega Optical, Brattleboro, Vermont) reflected the incident light from DISCO bending magnet onto the sample [18]. Emission was recorded with a Rollera XR CCD camera (Qimaging, Surrey, Canada) through bandpass filters at 510–560 nm (Omega Optical, Brattleboro, Vermont). Fluorescence images of vegetations were acquired at λ_exc_ of 275 nm with 1-s exposure.

### Fluorescence of ofloxacin in PBS and serum

The spectral microscope was also used to study the fluorescence of ofloxacin in PBS and rabbit serum. A 140 µmol/L solution of ofloxacin in PBS (pH 7.4) was deposited in glass cupules closed by quartz coverslips and examined at λ_exc_ of 275 nm. The same experiment was done with control and ofloxacin-treated rabbit sera to determine if ofloxacin fluorescence in serum correlates with its concentration.

### Spectral pre-processing and data analysis

Spectra were spike filtered and noise was corrected by a Fourier-Transform filter using a home made Matlab procedure (Marie-Françoise Devaux, Institut National de la Recherche Agronomique, Nantes, France). To compare control and ofloxacin spectra acquired from different maps, an offset was applied to all spectra in order to set the count at 288 nm to zero. Spectra with less than 100 counts at 339 nm were excluded in order to optimize the signal to noise ratio. Wavelengths superior to 540 nm were excluded in order to exclude the Rayleigh band harmonics. We compared control and ofloxacin-treated spectra after stratification on the fluorescence intensity at 339 nm - the main peak of fluorescence emission spectra, because fluorescence at 339 nm slightly varied between maps, due to experimental factors as slice thickness, microscope focusing and alignment, and photobleaching.

All map processing, spectra preprocessing and analysis were performed with Matlab R2007b and the SAISIR 2008 package of functions for chemometrics in the Matlab* environment (http://easy-chemometrics.fr). Fluorescence intensities were compared by the Mann-Whitney test.
